# Design of a “Cobot Tactile Display” for Accessing Virtual Diagrams by Blind and Visually Impaired Users

**DOI:** 10.3390/s22124468

**Published:** 2022-06-13

**Authors:** Satinder Gill, Dianne T. V. Pawluk

**Affiliations:** Department of Biomedical Engineering, Virginia Commonwealth University, Richmond, VA 23298, USA; dtpawluk@vcu.edu

**Keywords:** assistive technologies, visually impaired, haptics, admittance control, dead reckoning, tactile displays

## Abstract

Access to graphical information plays a very significant role in today’s world. Access to this information can be particularly limiting for individuals who are blind or visually impaired (BVIs). In this work, we present the design of a low-cost, mobile tactile display that also provides robotic assistance/guidance using haptic virtual fixtures in a shared control paradigm to aid in tactile diagram exploration. This work is part of a larger project intended to improve the ability of BVI users to explore tactile graphics on refreshable displays (particularly exploration time and cognitive load) through the use of robotic assistance/guidance. The particular focus of this paper is to share information related to the design and development of an affordable and compact device that may serve as a solution towards this overall goal. The proposed system uses a small omni-wheeled robot base to allow for smooth and unlimited movements in the 2D plane. Sufficient position and orientation accuracy is obtained by using a low-cost dead reckoning approach that combines data from an optical mouse sensor and inertial measurement unit. A low-cost force-sensing system and an admittance control model are used to allow shared control between the Cobot and the user, with the addition of guidance/virtual fixtures to aid in diagram exploration. Preliminary semi-structured interviews, with four blind or visually impaired participants who were allowed to use the Cobot, found that the system was easy to use and potentially useful for exploring virtual diagrams tactually.

## 1. Introduction

Graphic visual representations are increasingly used as the sole means to communicate a wide range of information at work, in school, and for daily living. Unfortunately, individuals who are blind and visually impaired (BVIs) currently have very limited access to the information in these graphics. Physical tactile graphics are the most common alternative used by BVIs to access this information, but they are often not available, are time-consuming to make, are expensive, bulky, and exhibit wear and tear relatively quickly. They can also be cumbersome in dynamic environments, such as when analyzing data or surfing the web, where rapid access to a large number of diagrams may be needed. However, BVIs’ independent access to information in diagrams is important to ensure their autonomy and provide equal opportunities for their advancement in education and employment. New methods are needed to address the problems of physical tactile graphics. In this paper, we focus on the design and development of a display device that focuses on a low-cost, rapidly refreshable method to display diagram information that is neither bulky nor suffers from wear and tear.

The use of alternate text descriptions is one such method that could address these issues. However, this method can often have difficulty communicating spatial concepts that are often key for understanding fields such as science and engineering (e.g., what is a sine wave, and what is meant by its phase). In addition, the ability for a BVI user to independently discover spatial patterns and relationships in graphical information is often lost as this step is part of the process of creating a summary word description (as, for example, the alternative of listing the raw locations of 100 data points would not be practical or easily comprehended). These concepts and tasks may be an essential part of a desired job or required school class. Finally, word descriptions can also be difficult to formulate for unfamiliar objects, especially for young children for whom the descriptions would need to fit within their limited vocabulary. It is unclear how one could explain to a child the concept of “above” without a physical demonstration or diagram.

Refreshable tactile displays/surfaces [1,2,3,4,5,6] have been proposed as alternatives, which allow for quick, dynamic access to electronic/virtual tactile diagram representations. At one end of the spectrum are tactile pin displays made from a large matrix of pins; however, these displays are very expensive despite covering much smaller areas compared to typical paper tactile diagrams and having limited spatial resolution (cost is an important consideration as most BVIs live below the poverty line). At the other end of the spectrum are surface haptic methods (e.g., vibration feedback on tablets). However, surfaces with vibration feedback are also limited in size (due to vibration strength) and provide only a single point of contact with a diagram (in contrast to the whole hand). The size of a display is a significant concern as even for those diagrams that have sufficient spatial resolution in their scaled-down form, reducing the size of the diagram has a negative effect on user performance [7]. The use of electrovibration could resolve the issue of size but is still restricted to a single point of contact with a diagram. Electrostatic displays can provide multipoint contact with a diagram, such as with the display created with specially instrumented gloves interacting with an LCD monitor [5]. However, this method cannot currently provide spatially distributed information within a finger, which is also important.

Small, mobile, refreshable pin displays with kinesthetic tracking [8,9] are more cost-effective than large matrix pin displays while enabling large workspaces through the movement of the hand/device across the virtual surface of the diagram. The motion of the hand with the pin display also allows these displays to resolve finer spatial details through temporal coding. The use of multipoint contact on the same and/or multiple fingers also significantly improves user performance compared to single point contact [10,11] and there can be no limitation in size as with surface displays. However, although a significant improvement in the ability to identify objects portrayed in diagrams occurred with multiple contacts, the time taken for a BVI user to explore the graphic was still much greater than for a physical tactile diagram [11]. This is problematic as it further exacerbates task completion time differences between sighted and blind users that exist due to differences in the information processing capacities of vision and touch.

Therefore, it is important to consider how the time needed to explore and understand a tactile diagram can be decreased while maintaining the aspects of previous small, refreshable pin display device designs that resulted in improved accuracy [8,11]. The main difficulty in using these types of displays [11,12] appears to be the inefficiency in which users track edges and lines. Simplifying diagrams so that a straight-line approximation is used for all edges and lines can make tracking edges/lines easier and improve performance [12]. However, this type of simplification is not always appropriate as the curvature of the edges and lines may be a key component of the information being relayed. It is also not clear how significant an improvement in response time occurs [12]. An alternate possibility is to provide guidance to the BVI user in the exploration process. Some researchers (e.g., [13,14]) proposed using the tactile display itself to provide symbols for navigational guidance, although this precludes providing information on the actual contact interaction with the diagram and is expected to be more cognitively demanding then someone (or something) guiding the hand physically.

In contrast, the use of guidance/virtual fixtures on haptic/robot force feedback devices (e.g., Phantom Omni) have been used for exploring simple line graphs and scatter plots, with both response time and accuracy better than with free exploration [15]. However, the algorithms have not been investigated for more complex diagrams or the potential benefits of integration with refreshable pin displays. In addition, the devices used have relatively small workspaces, are typically bulky, vary in cost from expensive to extremely expensive and, needlessly for 2D diagrams, operate in 3D space. A potentially better possibility would be a small, omnidirectional mobile robot, which could be small and lightweight, have a potentially arbitrarily large 2D workspace independent of the robot size, and be low in cost. Although a previous prototype was developed within our lab [16], it was bulky, had significant problems with position accuracy (critical for tactile diagram interpretation), and had not implemented any guidance/virtual fixtures on the device (including the needed measurement of the applied force by the user to allow shared control).

The focus of this paper is first to describe the development of a testbed device that combines a multifingered tactile pin display with a small and affordable mobile Cobot that can provide robotic assistance/guidance using haptic virtual fixtures in a shared control paradigm in a large workspace. This includes the development of a method to obtain sufficient position accuracy for the Cobot and tactile pins and the implementation of guidance/virtual fixtures that incorporates the user’s applied force. Second, the device is verified to have the functionality intended. In particular that: (1) sufficient position accuracy is achieved (this has been previously a problem for tactile mice and cofounded the interpretation of their use in accessing virtual diagrams), and (2) shared control with BVI users does not produce any unexpected outcomes (e.g., unexpected movement trajectories). The latter, as well as the usability of the device, is obtained through a preliminary user study and structured interviews with BVI users.

## 2. Previous Work

The haptic devices previously used to provide guidance and virtual fixtures for exploring tactile diagrams and teaching motor tasks, such as learning to write, have several limitations: they have relatively small workspaces, are typically bulky, vary in cost from expensive to extremely expensive and, needlessly for 2D diagrams, operate in 3D space. Furthermore, they are impedance type devices, which are usually low in inertia and are backdrivable. Unfortunately, being backdrivable force-source actuators can result in unexpected and undesirable movements of the devices [17]. It also does not easily allow shared control of the robot’s movement with the user. An admittance-type device would be a better choice as it does not suffer from these problems. One possible alternative is an admittance control device functioning similar to a 2D plotter. However, a 2D plotter design would be large and heavy for even a moderately sized workspace, as well as lack smooth motions in nonperpendicular directions. A possibly better admittance control device is a small, omni-wheeled mobile robot: this could be small and lightweight, have a potentially arbitrarily large workspace independent of the robot size, normally use admittance control, and achieve smooth motion in arbitrary directions due to the use of omni wheels.

A prototype of an omni-wheeled mobile robot, for this purpose, was previously developed in our laboratory [16]. The developed prototype allowed for a commanded input force to be decomposed into three distinct velocities using an admittance control model to produce 2D omnidirectional movements of various magnitudes. Unfortunately, there were some significant design issues with the protype. The physical dimensions of the device, including the main processor and omnidirectional wheels, were large and bulky, making the device hard for a user to maneuver with one hand. In addition, a method to sense the force applied by the user was not incorporated into the device and used to provide shared control of the robot. However, the most significant problem was that measurement of the x, y, and *θ* position of the robot (needed to determine its location in the virtual diagram) was very poor. This was because the position was measured at the rotary shaft encoders of the omnidirectional wheels, which differed significantly from the actual location of the mobile robot due to wheel slippage. This is problematic as wheel slippage is an inherent characteristic of omnidirectional wheels [18]. These design issues need to be resolved while keeping the cost of the device low, as most BVIs live below the poverty line.

Previous mobile tactile displays used a variety of low-cost methods to determine the position of the device. Many displays (e.g., the VT Player) used a mouse sensor to obtain position information. However, the x, y position information provided by these sensors is highly inaccurate for determining the haptic position of a device with respect to some starting point [19] and no angular orientation is given. The latter is important for mapping the pin locations of the tactile display onto the virtual diagram when only the device (i.e., sensor) location is measured. The alternative would be to keep track of the location of all the individual pins separately (with 16 pins in total).

Both graphics tablets with a 2D radio-frequency coupler on the tactile device and touchscreen displays have been used for more accurate haptic x, y position measurements (e.g., [19,20]), but they still do not provide angular position and also introduce a restriction on the size of the active area. A size restriction is particularly problematic for large diagrams such as maps and blueprints, where spatial continuity is important to facilitate the already difficult task of exploring these diagrams to determine spatial relationships (such as making short cuts from one point to another).

An alternative is to consider newer dead reckoning methods used in mobile robot applications. Bonarini et al. [21] showed that the problem of cumulative errors when using encoders on the device wheels for dead reckoning can be overcome using two optical mice attached at the bottom of a robot to accurately calculate robot pose. An alternate sensor fusion approach was successfully used in [22], where data from an optical mouse sensor, the yaw angle calculated from IMU data, and the wheel encoder data were combined using an extended Kalman filter to accurately estimate the position of a two-wheeled robot. Both methods showed promising results and were initially considered as potential position-sensing systems for our device. However, given the size and cost constraints of our device, these were not selected for implementation. This is because implementation of the first method requires a significant increase in the size of our device over other methods, due to the need to separate the two optical mice by a distance and still have them attached to the robot base. Although implementation of the second method [22] requires the use of only a single mouse sensor, the information gathered from this sensor needs to be combined with information from both an IMU and wheel encoders, resulting in increased system cost. Moreover, this method also requires that the mouse sensor be placed on the robot’s edge, resulting in increased size and complexity.

Here, we will consider a sensor fusion approach using a low-cost dead reckoning method that addresses these issues by using a single optical mouse sensor and a commercially available IMU. In addition, neither needs to be placed away from the center of the robot base. The displacement data provided by the optical mouse sensor is combined with the orientation angle (*θ*) from an IMU to determine the precise x, y location of the device, as well as its orientation.

In considering the form of the tactile feedback that should be provided to the BVI user, it should be noted that BVIs use their whole hand (and sometimes both hands) in exploring a tactile graphic [23]. This consists of spatially distributed information within a finger pad and across multiple fingers. Previous mobile tactile displays have used single-element vibration feedback for one finger (by vibrating the entire device, such as a touchscreen display, e.g., [24]) or multiple fingers (using finger-mounted devices that could freely move independent of each other [11,25]), or spatially distributed tactile feedback (pin matrix) on single or multiple finger pads a fixed distance apart [19,20].

As people use both spatially distributed information within a finger pad and across fingers when directly interacting with the environment, including both components seems warranted. The question is what is an appropriate number of tactile elements to have per finger pad and how many finger pads should be used. If we would solely consider the tactile resolution of the finger pads for determining the number of elements per finger pad, a 400-pin array would be required for each finger [26]. However, Weisenberger [10] showed that although increasing the number of tactile elements providing feedback to a finger improves performance, the largest improvement was between one and four elements. This suggests a more tractable number of pins for each finger on a mobile device. In terms of how many fingers to provide feedback for, Burch and Pawluk [11] showed that providing feedback to multiple fingers versus a single finger also significantly improved performance when textured diagrams (the most recommended tactile diagram format) are used. The most significant increase occurred between one and two fingers [25], which suggests that feedback for only two fingers is needed. The study also found that BVI users usually kept their fingers a comfortable distance apart and did not appear to change the distance between them (although they could), suggesting that a fixed distance with the fingers in a natural pose would work best. We will consider the use of pin displays for two fingers on the exploring hand of at least four elements each.

## 3. Materials and Methods

### 3.1. System Overview

**Figure 1:**
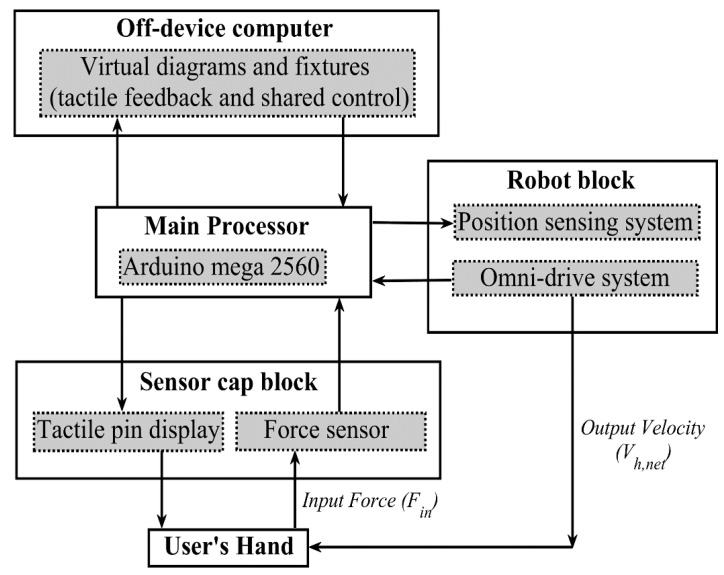
Block diagram illustrating the interaction between functional blocks of the system.

**Figure 2:**
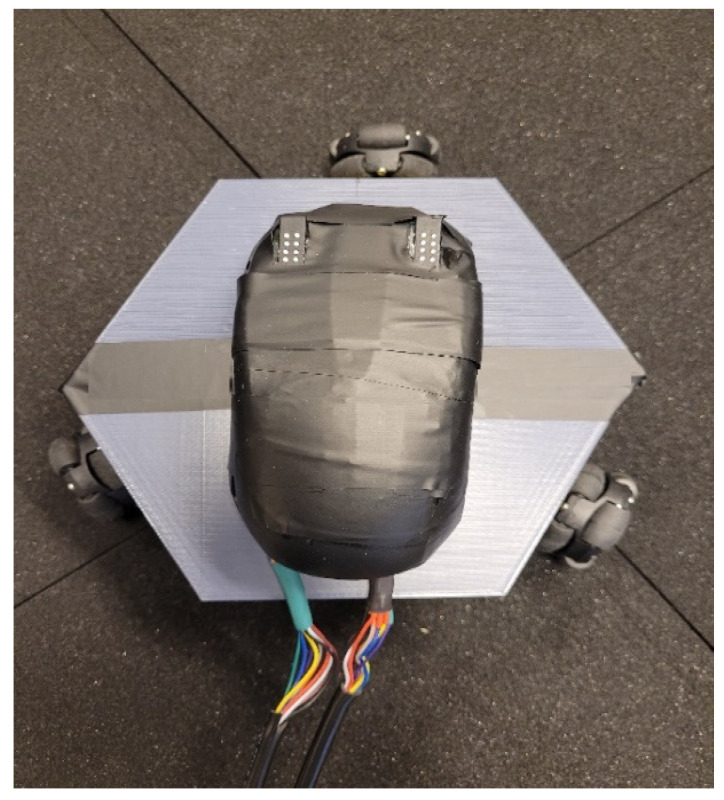
System current prototype.

The Cobotic tactile display acts as a refreshable display that both portrays a virtual diagram represented in electronic form and guides its haptic exploration. The system is made up of four functional blocks: the main processor, the mobile robot base (robot block), the cover shell containing the components which directly interact with the user (sensor cap block), and the higher-level software representing the virtual diagram and providing the shared control/virtual fixtures and tactile feedback signals (algorithm block). The interaction between the blocks, as well as their main components, is given in Figure 1. Figure 2 shows the system’s current prototype.

To haptically explore the virtual diagram, the user grasps the cover shell surface with one of their hands (with their index and middle fingers resting on the tactile pin display) and applies a planar force vector according to their desired exploration intentions. The entire force applied by the user is measured by a two-axis force-sensing system that connects the shell to the robot base. The applied force, together with the device’s planar position measured on the robot base, is sent to an off-device computer. The off-device computer then uses these measurements combined with the algorithm block to determine where the user is currently located in the virtual diagram. From the diagram location, the algorithm block provides the signals to control the tactile pin displays (implementing the tactile feedback) and the movement of the robot base (implementing the guidance/virtual fixtures). These signals are then sent to the main processor of the device to control the individual pins on the tactile display and the omni drive of the mobile robot base.

### 3.2. Main Processor

The main processor is a commercially available Arduino board (Mega 2560) that acts as the central component that controls device behavior and the primary communicator between different hardware components. The Arduino board is responsible for collecting and relaying inputs from the device (measured planar force and position) to the algorithm block on the off-device computer. It also relays the desired mobile robot commands (base velocity) and movement of each of the pins (on/off, vibration frequency) in the tactile pin display from the algorithm block, as well as controls the timing of all the outputs.

### 3.3. Robot Block

**Figure 3:**
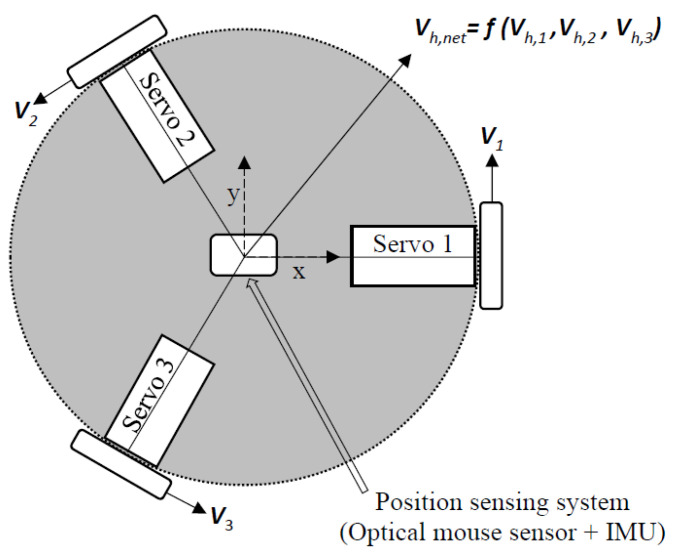
Diagram representing the robot block.

The robot block consists of an omni-drive system, which allows the mobile robot base to move directly in any planar direction, and a position-sensing system that measures the planar position of the center of the omni-drive platform and to which it is rigidly attached (Figure 3). The key requirements of this block include a cost-effective miniature design allowing for smooth and precise mechanical movement, and accurate position localization (x, y, and *θ*), in the 2D plane with respect to a start location. It is also desirable to have a large planar workspace (ideally large enough to display standard-sized (24″ × 36″) architectural blueprints of buildings (for navigation planning).

Additionally, a push button switch is housed on the side of the omni-drive platform to be used as a homing button, which moves the device to the heart of the diagram.

#### 3.3.1. Omni-Drive System

The omni-drive system is a motorized platform consisting of three servo motors (Hitec multiplex: HSR-2645CR) positioned concentrically 120° apart around the vertical axis of the base. Each servo motor is attached to a 38mm plastic omni wheel (Nexus: RB-Nex-136). Each wheel consists of small spinners placed around the radius of the main wheel frame and oriented orthogonally to the wheel’s axis of rotation. Due to this setup, the wheels can achieve smooth omnidirectional movements in a plane (x, y, *θ*). Using this design, the device can be moved linearly in the xy-plane and rotated around the *z*-axis freely.

The desired movement of the robot base is determined in the algorithm block, which provides the overall desired velocity vector for the robot base for that instant in time$\;V_{h,net}$. This velocity is determined from the admittance control equation (Section 3.5.2), which has inputs from the guidance algorithm and the force applied by the user. The omni-drive system then takes the net velocity $V_{h,net}$ and decomposes it into three velocity components $V_{h,1}$, $V_{h,2}$, and $V_{h,3}$ [27], one for each wheel. Each omni wheel comprises the main wheel frame and small free-rolling spinners oriented at 90° with respect to the main wheel frame. Therefore, the total linear velocity $V_{h,i}$ of each omnidirectional wheel can be represented as:

(1) $$V_{h,i}=\sqrt{\;\;\;V_{i}{}^{2}+V_{i,\;\mathrm{spinner}}{}^{2}}$$

where $V_{i}$ is the linear velocity of the wheel’s mainframe, $V_{i,\;\mathrm{spinner}}$ is the linear velocity of the free-rolling spinners, and $V_{h,i}$ is the total linear velocity of each omnidirectional wheel. The linear velocity of the main wheel frame, $V_{i}$, can be related to the angular velocity, $\dot{\theta }$, of its corresponding servo motor by:

(2) $$V_{i}=r\times {\dot{\theta }}_{i}$$

where r is the radius of the main omni-wheel frame. Using omnidirectional kinematics modeling it can be shown that:

(3) $${\dot{\theta }}_{1}=\frac{V_{1}}{r}=\frac{1}{r}\left[V_{hx}\right]$$

(4) $${\dot{\theta }}_{2}=\frac{V_{2}}{r}=\frac{1}{r}\left[-\frac{1}{2}V_{hx}+\frac{\sqrt{3}}{2}V_{hy}\right]$$

(5) $${\dot{\theta }}_{3}=\frac{V_{3}}{r}=\frac{1}{r}\left[-\frac{1}{2}V_{hx}-\frac{\sqrt{3}}{2}V_{hy}\right]$$

where $V_{hx}$ and $V_{hy}$ are the overall linear velocity of the robot base in the x and y directions as shown in Figure 3.

The HSRS-2645CR servo motors are controlled by commanded speed directly.

#### 3.3.2. Position-Sensing System

**Figure 4:**
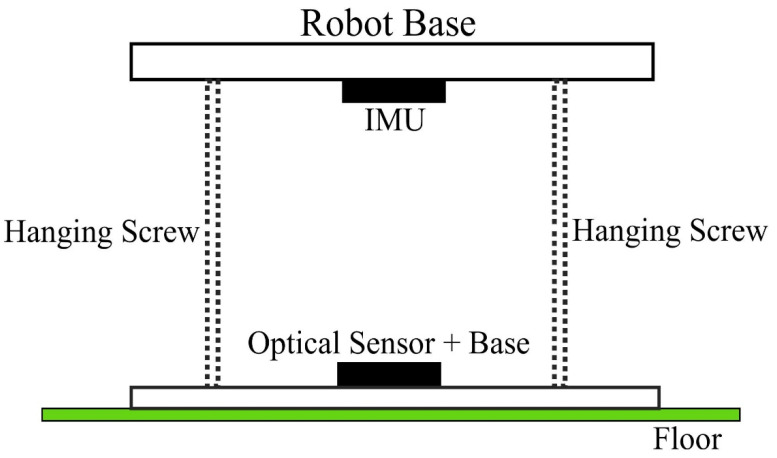
Composition of the optical mouse sensor and an inertial measurement unit coupled underneath the robot base.

The position-sensing system, which measures the Cobot’s planar position, plays a very critical role in the overall performance of the Cobot: reasonable precision of these measurements is needed to accurately depict the tactile diagram. The system consists of an optical mouse sensor (Pixart Imaging: PAW3515DB) coupled with a commercially available IMU (Sparkfun Electronics: MPU9250) to determine the precise location and orientation information of the device. The optical mouse sensor is coupled underneath the moving robot base using hanging screws to measure the movement of the floor with respect to the moving robot (Figure 4). The IMU is attached to the robot base as well (Figure 4).

The PAW3515DB optical mouse sensor is a low-cost 2D motion sensor that uses sequences of images captured at a frame rate of 3300 frames/s to estimate the relative motion between the sensor and the environment. Due to its high resolution of up to 1600 dots per inch, this sensor can provide accurate displacement along the *x*-axis (∆*x*) and *y*-axis (∆*y*) of the 2D coordinate system. We used a resolution of 1000 dots per inch. On the robot base, the xy-plane of the optical mouse sensor is aligned with the xy-plane of the omni-drive system (i.e., the device movement along the x-axis or y-axis corresponds to optical mouse sensor displacement along these axes as well). The displacement data (number of dots moved) from the optical mouse sensor is processed by the main processor to update the current position of the Cobot.

The MPU9250 is a 9-axis MEMS system that consists of two chips: the MPU6500, consisting of a 3-axis gyroscope and 3-axis accelerometer, and the AK8963, containing a 3-axis magnetometer. The MPU6500 provides linear acceleration and angular velocity information in the *x*, *y*, and *z* directions, whereas the AK8963 provides magnetic field information in these three directions. The *z*-axis of the omni-drive system is aligned with the *z*-axis of the IMU (i.e., in parallel with the gravitational acceleration). The angular velocity information from the gyroscope can be used to calculate the orientation angle ($\theta_{gyro}$) using the following equation:

(6) $$\theta_{gyro}\left(t\right)=\overset{t}{\underset{0}{\int }}\omega_{z}\left(\tau \right)d\tau$$

where $\omega_{z}\left(.\right)$ denotes the angular velocity along the *z*-axis. However, this is prone to long-term (low-frequency) integration errors due to the inherent bias in the gyroscope output.

The magnetometer can also be used to measure the orientation angle ($\theta_{mag}$), using the following relation:

(7) $$\theta_{mag}\left(t\right)=\left[\frac{m_{x}\left(t\right)}{m_{y}\left(t\right)}\right]$$

where $m_{x}\left(t\right)$ and $m_{y}\left(t\right)\;$ denote the earth’s magnetic field strength along the *x*- and *y*-axes. In contrast to the gyroscope measurement, this orientation angle is prone to short-term (high-frequency) errors due to hard-iron and soft-iron distortions in the magnetic field signals.

To resolve the limitations of the two methods for measuring the orientation angle, a complementary filter is used. The filter proposed has the advantage of being simpler, faster and requires less computational power than using a Kalman filter. The idea is that in the short term, we use $\theta_{gyro}$ because it is very precise and not susceptible to external forces. In the long term, we use $\theta_{mag}\;$ because it is not affected by bias errors. This idea can be formulated into the following equation to calculate Cobot’s orientation angle (*θ*):

(8) $$\theta^{k+1}=\frac{T}{T+\Delta T}\left(\theta^{k}+\Delta T\omega_{z}^{k}\right)+\frac{\Delta T}{T+\Delta T}\left(\theta_{mag}^{k}\right)$$

where T indicates the desired time constant, ΔT indicates the sampling interval, and {k, k + 1} denotes the time-indices of the discrete signals. Finally, the following equation was used to calculate the orientation angle *θ*:

(9) $$\theta^{k+1}=0.98\left(\theta^{k}+\Delta T\omega_{z}^{k}\right)+0.02\left(\theta_{mag}^{k}\right)$$

The orientation angle (*θ*) from the IMU calculation is used to adjust the displacement data (Δx and Δy) provided by the optical mouse sensor to obtain true displacement ($\Delta x_{true}$ and $\Delta y_{true}$) using the following equations:

(10) $$\Delta x_{true}=\Delta x\times cos\theta -\Delta y\times sin\theta$$

(11) $$\Delta y_{true}=-\Delta x\times sin\theta +\Delta y\times cos\theta$$

Although wheel slippage may occur, this is accounted for in the algorithm block by using the true position measured with the position-sensing system rather than the rotational angle of the motor.

### 3.4. Sensor Cap Block

**Figure 5:**
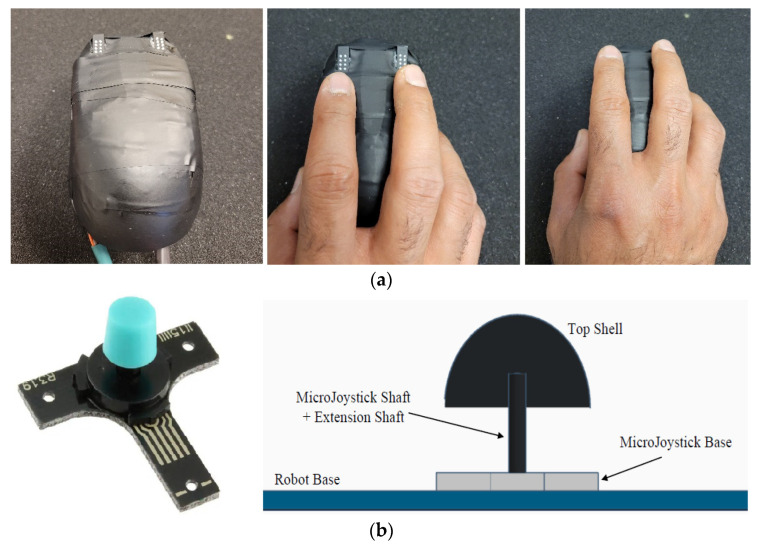
(**a**): Sensor cap block consisting of the top shell, force-sensing system, and tactile display; (**b**): MicroJoystick (left) and connection between the robot base, MicroJoystick, and top shell (right).

The sensor cap block (Figure 5) consists of a mechanical shell which is mounted as a cap on the force-sensing system (existing between the mobile robot base and the cap) and otherwise “floats” above the robot base. The shell provides a place for the user to hold the device and apply force, as well as to obtain tactile feedback through two electronic Braille cells mounted within the cap. The key requirements for the sensor cap block are that it is easy and comfortable to hold while the device moves, measures a reasonable approximation of the applied force by the user in the xy-plane, and provides tactile feedback to the user’s fingertips based on their location in the virtual diagram.

#### 3.4.1. Shell

The current prototype uses the top surface of a computer mouse due to its ergonomic design that makes it easy for a user to grasp and naturally rest their index and middle fingers on the top (where the tactile displays are located).

#### 3.4.2. Force-Sensing System

The force-sensing system uses a MicroJoystick (Interlink Electronics: 54-24451) to measure the applied force. It is essentially an isometric joystick that preserves an essentially fixed position relationship between the user’s hand and the robot base when force is applied to the stick. Four pressure-sensitive force-sensing resistors (FSRs) at its base measure the applied force’s magnitude. As the FSRs are each placed in a pattern corresponding to the cardinal directions ($FSR_{East}$, $FSR_{West}$, $FSR_{North}$, and $FSR_{South}$), with the output of each of these FSRs measured with respect to a common ground, they can be combined to approximate the vector force in the xy-plane (not including a scaling factor):

(12) $$F_{x}=FSR_{East}-FSR_{West}\;$$

(13) $$F_{y}=FSR_{North}-FSR_{South}$$

(14) $$\mathrm{Applied}\;\mathrm{force}\;F_{in}=\sqrt[{2}]{F_{x}+F_{y}}\;\tan^{-1}\frac{F_{y}}{F_{x}}$$

The precise scaling factor between the vector force$\;F_{in}$ calculated and the actual force is not needed as the algorithm using this information further applies an additional scaling factor that is to be tuned (Section 3.5.2).

#### 3.4.3. Tactile Display

The tactile display consists of two piezoelectric Braille cells (Metec AG: Braille cell P16), one for the index finger and one for the middle finger, mounted with the Braille cell pins (each a 2 × 4 pin array) extruding from the shell surface. The design follows that previously used for a passive, mobile tactile pin display extended to two Braille cells [20]. Each tactile pin is directly driven by turning power on and off through a corresponding solid-state single-pole double-throw relay (IXYS Integrated Circuits Division: LCC120). Each relay is directly controlled by an individual output line of the main processor, with all pins able to be controlled in parallel. The main processor is able to generate square waves of varying frequency (0–200 Hz) and duty cycle (0–100%) on each pin to create different vibration effects that produce a feeling similar to texture. The texture information is not intended to be interpreted as a particular texture, but instead used to help separate objects into their constitutive parts and indicate part orientation. Different textures indicate different parts and added “stripes” indicate orientation [11]. The texture to be produced by each pin at a given point in time is provided by the algorithm block based on the location of the pin within a textured virtual tactile diagram [11].

### 3.5. Algorithm Block

The algorithm block contains the representation of the virtual tactile diagram, which provides spatially coded information about the tactile (vibration) feedback and virtual haptic fixtures to be used. This information is used, along with the position and force measured by the device, to generate the signals controlling the individual pins on the tactile display and the omni-drive system. This block is implemented on a personal computer using MATLAB^®^.

#### 3.5.1. Spatial Tactile Feedback

For the tactile display, a tactile diagram is represented virtually as a colored image, where different colors correspond to the different frequencies used to generate textures and edges. The tactile diagram is translated from a visual diagram offline, using colors to separate parts of objects and represent their differing orientation, as this method was previously found to significantly improve performance [11]. The tactile feedback algorithm uses the current position of the center of the mobile base (x, y, *θ*), the geometry relating the center of the base to the location of an individual pin, and the scaling factor between the physical workspace and virtual diagram to determine the appropriate location of each pin in the virtual diagram. The vibration signal is then generated based on the color code of the corresponding diagram point. The use of *θ* as part of the calculation provides high position accuracy. In previous pin displays, users were required to keep the device vertical, distracting them from the primary task of exploring the diagram.

#### 3.5.2. Shared Control of Movement

To provide robotic guidance to our BVI users, guidance virtual fixtures were implemented along the edges of objects and object parts in the virtual diagram where we expect the most salient information about the object to be. Guidance fixtures facilitate movement in a preferred direction along the edges, which should make it easier for users to track edges and find other highly salient information to interpret the diagram. These guidance fixtures are represented in the computer as edges of the objects and object parts.

Control of the movement of the device is shared between the guidance virtual fixtures and the user. Although impedance control is often used with haptic feedback, being backdrivable force-source actuators, these systems are prone to instability. This can produce unexpected, undesirable movements when interacting with guidance/virtual fixture algorithms [17]. Instead, the system uses admittance control, which exhibits better behavior. An admittance controller uses the measured input force, provided by the user, along with an admittance variable, α, to produce the desired velocity, $V_{h,net}$, of the device, which is provided to the robot base.

(15) $$V_{h,net}=\alpha F$$

In absence of any virtual fixtures, F =$F_{in}$ (i.e., the force applied by the user to the device in the xy-plane). When a virtual fixture is present, the input force, $F_{in}$, from the user is decomposed (Figure 5a) into the force applied in the desired direction, $F_{d}$, and the undesired direction, $F_{\tau }$, (orthogonal to the desired direction) based on the position of the virtual fixtures on the virtual tactile diagram. The relative contribution of each of these forces is weighted using an admittance value, $k_{\tau }\;\epsilon \;\left[0\;1\right]$, to allow different relations in sharing control between the user and the fixture.

(16) $$V_{h,net}=\alpha \left(F_{d}+k_{\tau }F_{\tau }\right)\;$$

If the admittance value is set to zero, a virtual fixture completely constrains the motion to the preferred direction only (in our case, moving towards the closest edge). If the admittance value is set to one, the compliance of movement is equal in all directions, allowing the user to freely move. For a case when the admittance value is set to values between 0 and 1, the virtual fixture responds to the user input force and direction by encouraging desired motions along the path; however, it also allows a user to “break free” and explore other areas of the virtual diagram. This balance is expected to be important, as although our goal is to improve the ability of user’s to track edges, active, free exploration is considered an important aspect of haptic perception [28]. This suggests that an admittance value somewhere between 0 and 1 may be the best choice to obtain both objectives.

**Figure 6:**
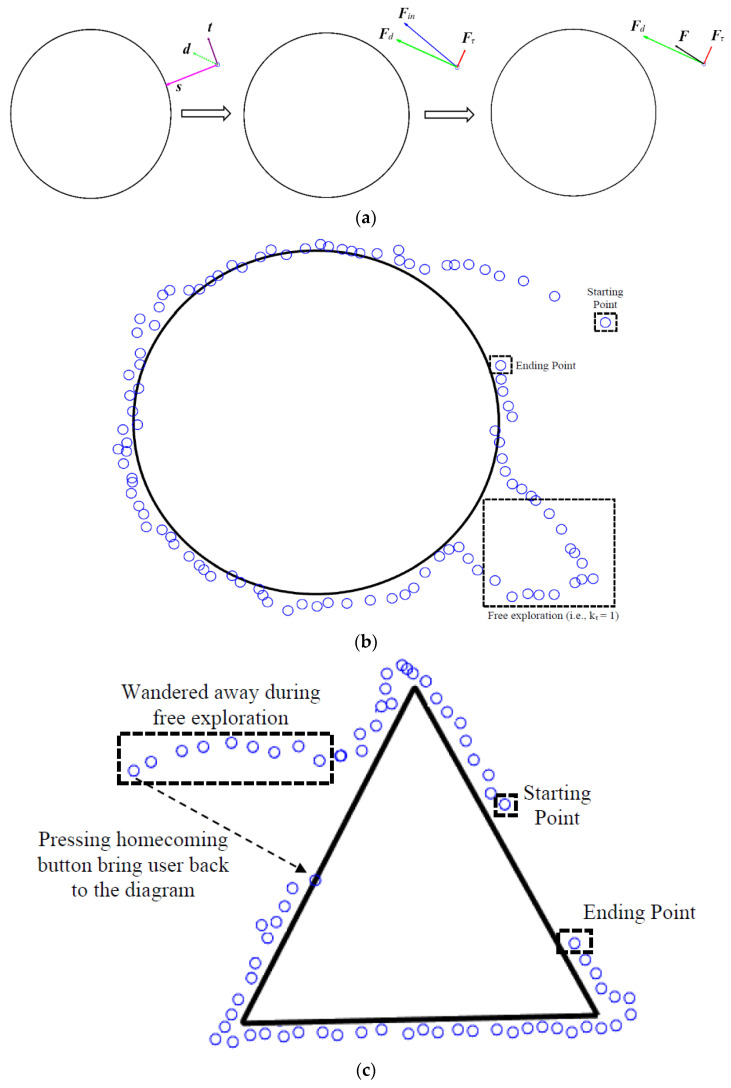
(**a**): Shows an illustration of a sample virtual diagram (circle) and the calculation of the final force depending on the location of the mobile robot and applied force. Here, both the admittance value ($k_{\tau }$) and stiffness factor ($k_{s}$) are set to 0.5. (**b**): Shows an illustration of a virtual object (circle) and the path followed by the mobile robot in response to a user’s input force. Here, the stiffness factor ($k_{s}$) is set to a value of 0.5. The admittance value ($k_{\tau }$) is set to 0.25 for all locations (i.e., to promote movement along the desired direction) except for locations marked with the dotted square (free exploration, $k_{\tau }$ = 1). (**c**): Shows an illustration of an equilateral triangle and the path followed by the mobile robot in response to a user’s input force. Use of homecoming button brings user back to the nearest point on the diagram. Blue circles show position of the robot w.r.t to the diagram.

In Figure 6, we use different admittance values between (0 $\leq k_{\tau }\leq 1$) for two different basic geometric shapes. This method first determines a shortest deviation vector, s, as a vector originating from the device’s current position, $p_{c}$, and pointing towards the closest virtual fixture (i.e., edge) point, $p_{v}$, on the virtual diagram.

(17) $$s=p_{v}-p_{c}$$

Thereafter, the desired direction (D) is calculated as the sum of two vectors: vector s and vector t. Vector t points in the direction of the tangent to the virtual fixture/edge at the closest point on the diagram.

(18) $$D=t+k_{s}s$$

(19) $$d=\frac{D}{∥D∥}$$

where $k_{s}$ is the stiffness factor for the virtual fixture which determines the contribution of the tangent and the shortest deviation to the desired direction D. d indicates a unit vector in the desired direction. Lastly, the desired force $F_{d}$ is calculated by projecting the input force $F_{in}$ onto the desired direction d. The nondesired force $F_{\tau }$ is calculated as the difference between the input force $F_{in}$ and the desired force $F_{d}$.

(20) $$F_{d}=F_{in}\times d$$

(21) $$F_{\tau }=F_{in}-F_{d}$$

If the user wanders away from the virtual diagram, the homing button can be used to move the Cobot to the nearest point on the diagram. Under this scenario, the shortest vector s (Equation (17)) is used to calculate the device-desired velocity such that ($V_{h,net}=\alpha s)$.

(22) $$V_{h,net}=\alpha \left(s\right)\;$$

### 3.6. BVI User Study Protocol

One-on-one semi-structured interviews were conducted with BVIs to obtain feedback as to the ease of use and utility of the developed device for exploring tactile diagrams. There were four participants; three of them had light sensitivity only whereas one had low vision (and was blindfolded when interacting with the device). In the interview session, the experimenter first explained the purpose of the device, the concept of a Cobot and shared control, and how to interact with the provided prototype. The experimenter then guided a participant’s hand in exploring the Cobot and work area. When the participant was comfortable with the device, they were given time to use it to interact with virtual tactile diagrams.

**Figure 7:**
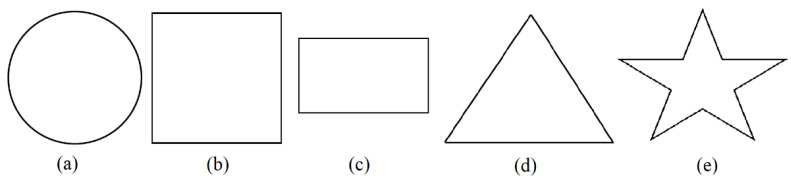
Two-dimensional geometric shapes. (**a**): illustration of a circle (diameter = 10.16 cm). (**b**): illustration of a square (length = 10.16 cm, width = 10.16 cm). (**c**): illustration of a rectangle (length = 10.16cm, width = 5.08 cm). (**d**): illustration of an equilateral triangle (side = 10.16 cm). (**e**): illustration of a star (height = 10.16 cm).

Each participant was allowed to use the Cobot to explore five different (in random order) 2D geometric shapes (Figure 7). Geometric shapes were chosen due to their simplicity and the frequency that they are used by BVI students. These virtual shapes were implemented using MATLAB such that their maximum span was 4 inches (i.e., 10.16 cm). The unit of measurement for these virtual shapes was set as ‘points’ (one inch was represented using 72 points), whereas one inch of mouse sensor movement was represented by 1000 dots. Therefore, a distance mapping factor of 0.072 (72/1000) was required. The following equation was used for the mapping:

(23) $$V_{d}=S_{c}\times 0.072\times C_{d}$$

where $V_{d}$ represents distance travelled on the virtual diagram, $C_{d}$ represents the distance travelled by the Cobot (mouse sensor data), and $S_{c}$ represents the scaling constant. The scaling factor $S_{c}$ is used to allow a diagram to span the available workspace within the user’s reach. During this experimentation a scaling factor of 0.5 was used (i.e., 2 inches of movement of the Cobot corresponded to one inch of movement on the virtual diagram).

The starting planar position (xy-plane) of the Cobot was set relative to the virtual shape and was always selected to be outside the diagram. At the starting point, the user’s hand is assumed to be in neutral position, which will define the orientation of the *x*- and *y*-axes; therefore; the initial rotation of the Cobot (*θ*) relative to the virtual diagram was set to zero. The algorithm for the admittance controller used three separate admittance values ($k_{\tau }$ = 0, $k_{\tau }$ = 0.5, and $k_{\tau }\;$= 1) for the same geometric shape so that users could feel the range of how the control of movement could be shared. The value of the stiffness factor ($k_{s}$) was set to 0.5 and not changed during the session.

Each participant was asked the following seven questions (Table 1) about their experience related to the usability of the Cobot. These questions were asked during the later stages of a participant’s exploration with the device. Participants were asked to rate their feelings of usability on a scale of one to five, with a value of five indicating strongly agree. They were also asked to share any specific comments related to these questions.

After each participant finished using the Cobot, they were also asked to provide feedback on their perceived usefulness of the shared control concept for diagram exploration both on its own and in combination with tactile feedback.

## 4. Results

**Figure 8:**
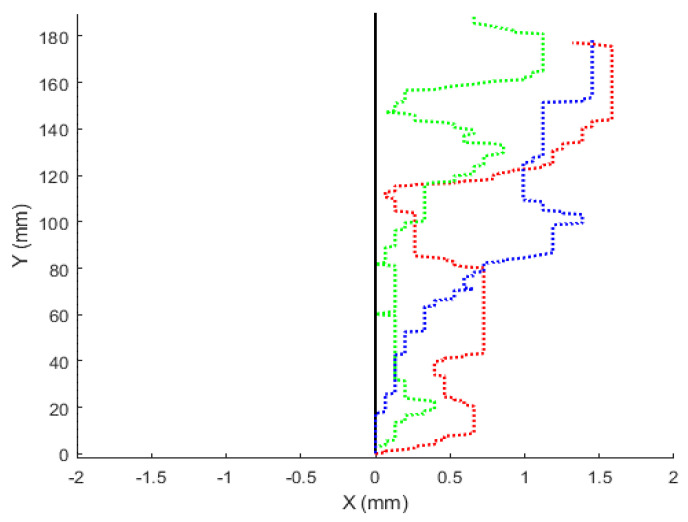
Robot movement along a simple trajectory (solid black line indicates the ideal trajectory; dashed red, blue, and green lines indicate trajectories followed by the robot in three different trials).

To validate the reliability of the mouse sensor data to represent the location with respect to the starting location, we tested the robot movement along a simple trajectory in various directions. Results are shown for the robot moving from the starting location to a 180 mm (7.1 inches) distance along the *y*-axis (i.e., [0,0] to [0,180]). The commanded trajectory is denoted by a solid (black) line in Figure 8. The results for three different trials of robot movement are shown (trial 1—dotted red line: [0,0] to [1.32,177.1]; trial 2—dotted green line: [0,0] to [0,184.59]; trial 3—dotted blue line: [0,0] to [1.45,179.5]).

For the user study, participants were observed in their initial interaction, the time it took to learn the system, and, after they learned the system, any problems or awkwardness in using the Cobot. All four participants quickly learnt how to use the Cobot and showed no signs of awkwardness or difficulty in using the device in the different shared control modes. Table 2 lists the rating scale answers provided by the four participants, P1, P2, P3, and P4, to the seven questions.

In terms of usability (question 1), all four participants stated that the device was small, maneuverable, and easy to use. Participant P4 mentioned that “the device would be more ergonomic (i.e., reduce fatigue during longer interaction times) if lowered in height”. This was consistent with observations by the study investigator of the interaction of the participants’ hands with the Cobot. It was observed that participants were not able to rest their arm on the desk while holding the top shell. Lowering the height of the top shell by approximately 1 inch would allow this to occur for all the participants.

In terms of question 2, all four participants further suggested that although the size was not a problem when using the device, it would be more portable if it was made smaller (easier to carry around).

For question 3, all participants liked the idea of being able to adjust the weighting (i.e., admittance value $k_{\tau }$) of how control was shared. They had no particular opinion towards using a particular fixed value of $k_{\tau }.$ Instead, all were of the view that being able to control the admittance value would provide them flexibility in interacting with the virtual diagram. For example, participant P4 suggested using stronger Cobotic guidance (smaller admittance value) when exploring newer diagrams or getting an overview of a diagram. On the other hand, they suggested having weaker Cobotic guidance to “break free” when they had general familiarity with the diagram and wanted to obtain more details. Participant P2 stated that “everyone is different, so the ability to control $k_{\tau }$ is a good thing”. All four participants thought that using a simple control knob on the Cobot to adjust the weighting would be easiest to use.

When asked about question 4, all participants agreed that it would be easy for most people to learn how to use the device. Participant P1 further suggested that “providing audio instructions on how to use the device would be helpful”.

All participants were enthusiastic about the homing button (question 5). They believed that this would be particularly helpful during free exploration. For example, if someone wandered away from key areas during free exploration and felt lost, the homing button could be used to move the device back to the heart of the diagram.

All four participants were of the view that the device was easy to use (question 6) and thought that the use of the mouse cap to control the robot was helpful (question 7).

In terms of usefulness, all four participants liked the idea of using Cobotic assistance when exploring tactile diagrams and did not report any problems when interacting with the device. Participant P4 stated that “this concept may be very helpful and they look forward to see how this can be used in different applications”. Similarly, participant P3 stated that “they would love to use the device” and participant P2 mentioned that “use of tactile feedback would be great help”.

## 5. Discussion

This paper describes the development of a small, cost-effective Cobot tactile display that provides distributed tactile feedback to two fingers, as well as the ability to provide haptic guidance to BVI users exploring a tactile graphic. The new prototype is reduced in size compared to the previous prototype and able to achieve higher accuracy in a position relative to a start location. The current position accuracy is sufficient for exploring a tactile diagram haptically without vision. The size of the workspace is potentially limitless, although restricted by practical limitations of arm reach. The new prototype also successfully implements the control/guidance of the Cobot based on the sensed force applied by the user and the guidance/virtual fixtures represented virtually in the off-device computer. Semi-structured interviews involving four BVI participants suggested the device is easy to use and has the potential to improve access to tactile diagrams.

Previous problems with position accuracy were overcome by the development of a dead reckoning module combining information from an optical mouse sensor and an IMU. Accurate haptic spatial perception is crucial in the absence of vision for correct interpretation of shapes and spatial relations in tactile diagrams [19]. Unlike the system used by Rastogi and his colleagues [19], our current prototype does not suffer from errors due to rotation of the user’s hand.

The current prototype also successfully implements guidance/virtual fixtures in conjunction to the admittance control of the movement of the robot base with shared control with the user, which was not completed in the previous version of the device [16]. This included the measurement of planar force applied by the user to the Cobot device to indicate intention of movement. Although angular movements by participants holding a wrist manipulandum can have a relatively small standard deviation of 2 degrees in matching tasks under controlled conditions [29], several reasons suggest that the measured force vector does not need to be as accurate for our purposes: (1) the force measurement does not affect the location accuracy of the tactile feedback or virtual fixtures, (2) the accuracy of “movement intention” is unlikely to be as high as under controlled conditions, particularly as the main focus of the user will be on interpreting the tactile/haptic feedback as a function of position, (3) the shared control algorithm is sufficiently complicated that users will not be able to predict the results of their “movement intention” in a highly precise manner, and (4) participant feedback from our user study indicated that users did not experience any negative effects when exploring diagrams.

The preliminary feedback from potential BVI users further suggests that the addition of guidance/virtual fixtures and shared control has the potential to improve access to refreshable tactile graphics, especially for diagrams that are more complex. Previous work by Paneels and his colleagues [15] suggested that using guidance/virtual fixtures alone may be sufficient, although they only examined performance with simple line graphs and scatter plots. The advantage, if this is true, would be a simpler and cheaper device. A comparison between the use of tactile feedback + guidance/virtual fixtures and the use of guidance/virtual fixtures only is needed to determine if their results extend to more complicated diagrams.

Originally the use of shared control with haptic fixtures also suggested that further study would be needed to determine the optimum settings for use of the Cobot. However, participants suggested and had a preference to use a simple knob so that they could control the amount of shared control themselves while actively exploring a diagram. This is likely to have great utility as the amount of shared control desired is likely to shift during the exploration of a diagram as the user’s intention shifts, as mentioned by one of the participants. For example, when using physical tactile diagrams in classroom settings, teachers of the blind will typically guide the BVI student over the whole graphic for an overview and show them any diagram keys. Then the student might be expected to more independently explore the graphic [30]. However, these steps may differ if the student is already familiar with the graphic.

## 6. Limitations and Future Work

The limitation of this work is that it was unable to draw any conclusions about the performance of BVI users with this device in terms of their ability to comprehend diagrams, the time taken, and the cognitive load. This was because the focus was on the assessment of the basic functionality of the system, in terms of position accuracy and the implementation of the shared control algorithm, and on user assessment of the usability and usefulness of the device. This is an important first step to ensure any user studies will produce meaningful results. Future studies will compare the use of the system to the use of haptic fixtures and shared control only, to the use of tactile feedback only, and to the current standard physical diagrams. Participants will be assessed in their understanding of the diagrams, the time taken to explore the diagram, and the cognitive load involved.

A limitation of the overall project is that it only focuses on the display aspect of providing diagram information. Another important aspect is the conversion of visual diagrams to tactile diagrams, which is a time-consuming task. The conversion process also involves a great deal of simplification that requires an experienced professional to create. However, this component can and is being addressed separately.

Other limitations of this work are that the user study and preliminary semi-structured interviews were conducted with a small number of BVIs, only simple diagrams were used, and a limited number of shared controlled levels were experienced. However, the participants were a random sample of the target population and their responses indicated an ability to extrapolate to more complex diagrams and differing levels of shared control.

## 7. Conclusions

The developed Cobot tactile display prototype was able to accurately render tactile feedback and guidance fixtures for the exploration of tactile graphics by BVIs. The display achieved both sufficient position accuracy and a large workspace. Participant feedback in a user study suggested that the device would be more comfortable if it was lowered in height, so that users could rest their arms on the table, and also reduced in size. Participants also liked the use of a homing button, which was also consistent with advice to have a reference point when exploring tactile graphics [30]. Further research is needed to determine how best to integrate guidance/virtual fixtures with tactile pin display feedback, or whether either alone performs better. The shared control paradigm also has potential benefits for BVI student–teacher interactions and other collaborations in remote learning and working environments.

## Figures and Tables

**Table 1:** List of questions related to usability of the Cobot.

Question Number	Question
1	The device was maneuverable
2	The device was too big to use effectively
3	The control knob was useful to control different admittance value settings $k_{\tau }$ (i.e., comparative strength to which they and the Cobot would share control)
4	Most people would learn to use this system easily
5	The homing button was useful to move the device to the heart of the diagram
6	The Cobot was easy to use
7	Applying force to the mouse cap was useful to control the Cobot

**Table 2:** Table listing rating scale answers to questions listed in Table 1.

Question Number	P1	P2	P3	P4
1	3	5	5	4
2	2	1	2	2
3	5	5	5	5
4	4	5	5	5
5	3	5	5	5
6	4	4	5	5
7	4	5	4	5

## Data Availability

Supporting data can be requested via email to the authors.
